# Comparing different types of statins for secondary prevention of cardio-cerebrovascular disease from a national cohort study

**DOI:** 10.1371/journal.pone.0247419

**Published:** 2021-02-25

**Authors:** Joungyoun Kim, Hyeong-Seop Kim, Yun-Jong Bae, Hyeong-Chul Lee, Jae-woo Lee, Hee-Taik Kang

**Affiliations:** 1 Mo-Im Kim Nursing Research Institute, Yonsei University College of Nursing, Seoul, Republic of Korea; 2 Department of Information & Statistics, Chungbuk National University, Cheongju, Chungbuk, Republic of Korea; 3 Department of Family Medicine, Chungbuk National University Hospital, Cheongju, Chungbuk, Republic of Korea; 4 Department of Family Medicine, Chungbuk National University, College of Medicine, Cheongju, Chungbuk, Republic of Korea; German Centre for Neurodegenerative Diseases Site Munich: Deutsches Zentrum fur Neurodegenerative Erkrankungen Standort Munchen, GERMANY

## Abstract

Statins have been recommended for use in atherosclerotic cardio-cerebrovascular disease (CCVD). The purpose of this study was to investigate the efficacy of five different types of statin in the secondary prevention of CCVD in patients. This study retrospectively designed and analyzed data from the National Health Insurance Service-National Health in Korea. Participants aged 40 to 69 years were categorized into five statin groups (atorvastatin, rosuvastatin, pitavastatin, simvastatin, and pravastatin). The primary composite outcome was defined as recurrence of CCVD or all causes of death. Cox proportional hazard regression models were adopted after stepwise adjustments for confounders to investigate the difference in efficacy among the different statins. Of the 755 final participants, 48 patients experienced primary composite outcomes. After adjustments, the hazard ratios (95% confidence intervals) for primary composite outcomes of atorvastatin, pitavastatin, and rosuvastatin groups were 0.956 (0.456–2.005), 1.347 (0.354–5.116), and 0.943 (0.317–2.803), respectively, when compared with the simvastatin group. There were no significant differences between the statins in efficacy for preventing recurrence of CCVD events and/or death in CCVD patients.

## Introduction

According to the World Health Organization fact sheet from 2017, atherosclerotic cardiovascular disease (ASCVD) is the number one cause of death worldwide. In addition, approximately 17 million people died from ASCVD in 2016, accounting for 31% of all global deaths. Of these deaths, 85% were reported to be due to a heart attack and/or stroke [1]. In Korea, the socioeconomic burden of cardio-cerebrovascular disease (CCVD) is rapidly increasing. CCVD is the second leading cause of death in Korea and accounted for one-quarter of total deaths in 2016 [2].

Strategies to prevent CCVD have important implications for substantially reducing mortality and related public health burdens. Dyslipidemia is the most important controllable risk factor for atherosclerotic CCVD. According to several previously published cholesterol guidelines, statins are widely administered for primary and secondary prevention treatments of atherosclerotic CCVD in individuals with dyslipidemia [3–6].

Various types of statins have been developed and approved for clinical use. Although most statins share common mechanisms of action, their pharmacokinetics and dynamics differ, and the clinical efficacy for improving patient lipid profiles and preventing ASCVD is unknown among the different statins [7]. Additionally, in Korea, there is a lack of evidence on the efficacy of each statin for the secondary prevention of CCVD compared with other statins.

The purpose of this study was to investigate the relationship between the use of five different types of statins (atorvastatin, rosuvastatin, pitavastatin, simvastatin, and pravastatin) and the composite outcomes (all causes of death and/or CCVD events) in CCVD patients using the national cohort data.

## Materials and methods

### Data source and study population

Data from the Korean National Health Insurance Service–National Health Screening Cohort (NHIS-HEALS) database, that was created based from the national health screening examinations between 2002–2003, was used. The NHIS-HEALS cohort consists of 514794 persons and represents approximately 10% of the 5.15 million eligible national health insurance subscribers aged 40 to 79 years who have undergone national health screening examinations. All the study participants received at least one health screening between January 2002 and December 2003. The participants in the database received a follow-up health examination and submitted a lifestyle and behavior survey, which included information on age, sex, socio-demographic factor, medical record, and laboratory results. A detailed description of the study design and methods was previously published [8].

**Fig 1:**
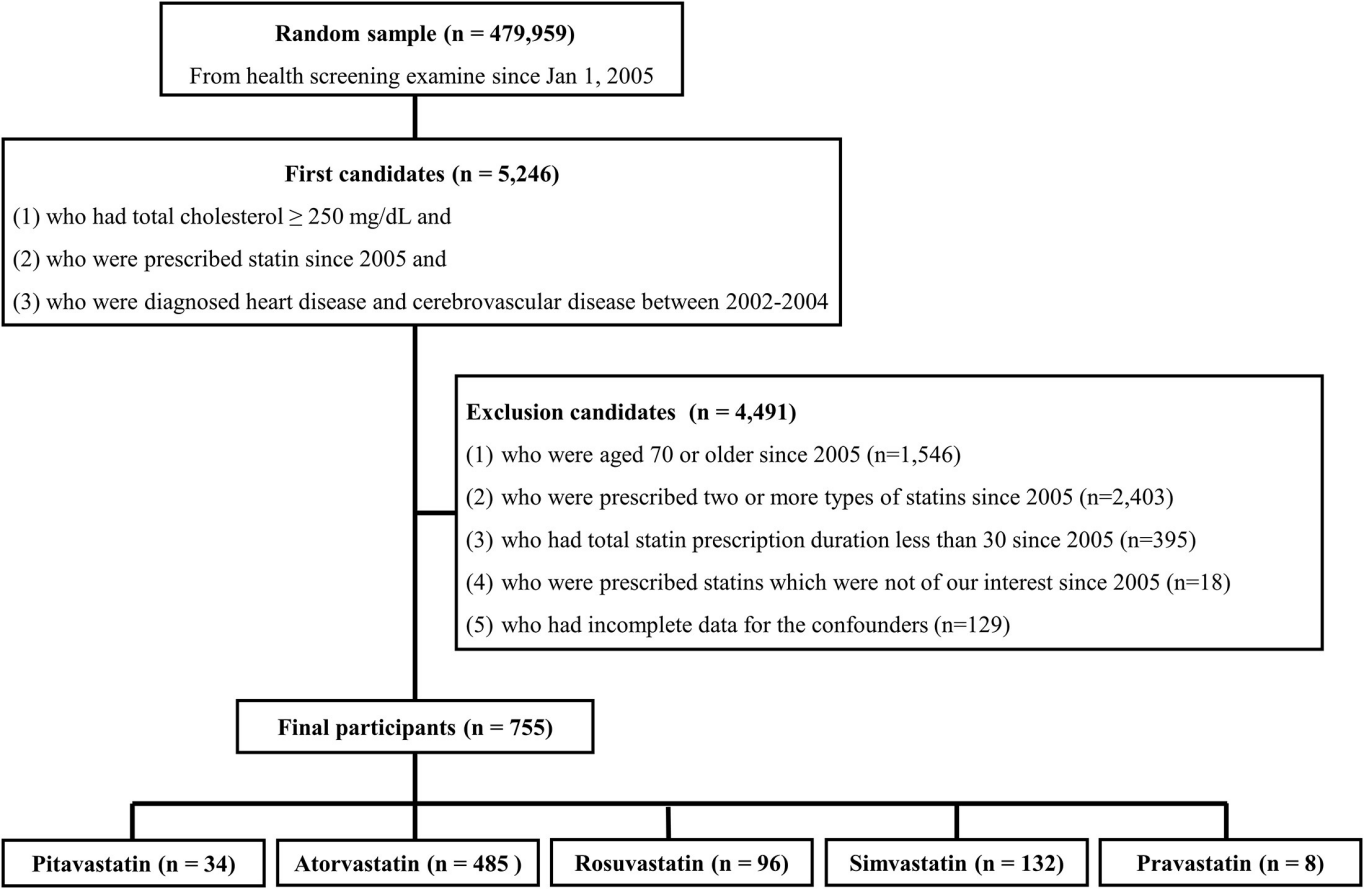
Flowchart of inclusion and exclusion criteria for participant selection method.

The participants in this retrospective study were selected from the NHIS-HEALS cohort (Fig 1). First, subjects were selected based on whether they had attended a health screening since 2005 (*n* = 479,959). Next, participants were selected only if they satisfied all the following conditions: (1) total cholesterol of ≥250 mg/dL, (2) been prescribed a statin since 2005, and (3) diagnosed with CCVD between 2002 and 2004 (*n* = 5246). To maintain a homogeneous participant pool, subjects were excluded according to the following criteria: (1) prescribed two or more types of statins since 2005 (*n* = 2403), (2) prescribed a statin for ≤30 days since 2005 (*n* = 395), (3) prescribed statins which were not one of our target drugs since 2005 (*n* = 18), and (4) participants with missing data in the confounding values criterion between 2005 and 2008 (*n* = 129).

To evaluate the differences among the different types of statins, the subjects were divided into five groups: atorvastatin, pitavastatin, simvastatin, rosuvastatin, and pravastatin. Other types of statins were not considered due to their small sample population. All participants took only one type of statin in this study. Of a total of 5246 participants, 4491 subjects were excluded. Finally, 755 individuals were included in this analysis (Fig 1).

This study was followed the guidelines of the Declaration of Helsinki (1975) and was approved by the Chungbuk National University Hospital Institutional Review Board (CBNUH-2019-07-013-001). Informed consent was waived as NHIS-HEALS were anonymized at all stages including during data clearing and statistical analyses.

### Operational definition

In this study, we defined CCVD patients as having one or more of the primary diagnostic codes, I20–I25 and I60–69, from the 10^th^ edition of the International Classification of Disease codes (ICD-10^th^ codes). The primary composite outcome in this study was defined as the recurrence of CCVD with ICD-10^th^ codes I21-24, I63, I65, and I66 and/or all causes of death since 2005. For further analysis, CCVD events were considered as secondary outcomes.

### Study period

The start date of the study was the first diagnosis date of CCVD. For subjects who had recurrence or death, the end date was the first recurrence date of CCVD or the date of death since 2005, whichever occurred first. For the remaining subjects, the study end date was the last date of the following events: (1) the most recent date of follow-up health screening, (2) the most recent date of a hospital visit, or (3) the most recent date of statin administration.

### Potential confounders

In this study, the following variables were considered as confounding variables: age, sex, body mass index (BMI), systolic blood pressure (SBP), glucose, total cholesterol, alanine aminotransferase (ALT) levels, a history of diabetes mellitus (DM), smoking status, alcohol consumption, physical activity, and economic status. These confounding variables were obtained from the records of the first health screening examinations. A history of DM, smoking status, alcohol consumption, physical activity, and economic status were classified as categorical variables; the remaining confounding variables were classified as continuous. The categorical variables, except for economic status, were extracted from self-reporting questionnaires and were recategorized for statistical analysis purposes: participants who answered “Yes” to “Have you ever been diagnosed with diabetes?” were classified as “DM”; smoking status was classified into two groups: “ever smokers” and “non-smokers”; alcohol consumption was divided into three groups: “rare” (less than twice a month), “sometimes” (twice a month to twice a week), and “often” (more than twice a week); physical activity was classified into three groups: “rare” (individuals who did not exercise), “sometimes” (exercise between 1 and 4 days per week), and “regular” (exercise more than 4 days per week); economic status was categorized into three groups: “low” (≤30^th^ percentile), “middle” (>30^th^ to ≤70^th^ percentile), and “high” (>70^th^ percentile).

### Statistical analysis

Continuous variables are expressed as mean ± standard deviation, while categorical variables are expressed as a percentage of the cohort. To evaluate the differences among the different types of statins, analysis of variance and Fisher’s exact tests were used. Kaplan-Meier methods and log rank test estimates were used to compare the prevention of CCVD effects by individual statin types. Cox proportional hazard (Cox-PH) models were performed to estimate the hazard ratios (HRs) for primary composite outcomes. In this study, the Cox-PH models were performed at three levels: model 1: age; model 2: age, smoking status, alcohol consumption, and physical activity; and model 3: all variables in model 2 and included a history of DM, economic status, BMI, SBP, ALT, and total cholesterol. All statistical tests were two-sided, and p-values were defined as statistically significant if they were less than 0.05. The statistical package, SAS Enterprise Guide version 7.1 (SAS Inc., Cary, NC, USA), and R (R Core Team, Vienna, Austria) were used to perform the analyses in this study.

## Results

Of the 755 final participants in this study (485: atorvastatin, 34: pitavastatin, 8: pravastatin, 96: rosuvastatin, and 132: simvastatin), 48 patients experienced the primary composite outcome during the study duration and accounted for 6.36% of the study population. The median follow-up duration was 12.4 years.

The baseline characteristics among five different statin groups are summarized in Table 1. All variables considered for this study were not significantly different among the five different statin groups. Although not statistically significant, individuals treated with simvastatin were the oldest, while patients in the pravastatin group were the youngest (Table 1). Total cholesterol levels were increased in pravastatin group and decreased in the rosuvastatin group. The prevalence of DM was ≥ 15% across all statin groups.

**Table 1:** Baseline characteristics according to statin type.

	Atorvastatin	Pitavastatin	Rosuvastatin	Simvastatin	Pravastatin	p-value
Number of patients	485	34	96	132	8	
Age, years	55.4 ± 6.6	55.0 ± 6.5	54.3 ± 6.2	56.5 ± 7.1	52.3 ± 6.9	0.081
BMI, kg/m^2^	24.8 ± 2.8	23.8 ± 2.6	24.0 ± 3.1	24.6 ± 2.9	24.6 ± 3.9	0.060
SBP, mmHg	126.4 ± 16.0	131.9 ± 17.0	124.8 ± 15.8	125.9 ± 15.8	126.3 ± 19.7	0.267
Glucose, mg/dL	96.5 ± 22.8	95.8 ± 15.5	96.2 ± 17.1	100.4 ± 31.0	94.4 ± 20.9	0.522
Total cholesterol, mg/dL	235.7 ± 50.1	240.1 ± 32.2	230.9 ± 32.4	240.7 ± 45.8	248.8 ± 27.7	0.504
ALT, IU/L	25.4 ± 14.8	22.5 ± 8.9	28.1 ± 26.6	28.5 ± 18.4	21.3 ± 13.2	0.151
DM, N (%)	99 (20.4)	10 (29.4)	15 (15.6)	39 (29.5)	2 (25.0)	0.066
Ever smokers, N (%)	101 (20.8)	7 (20.6)	25 (26.0)	33 (25.0)	1 (12.5)	0.669
Alcohol consumption, N (%)						0.759
Rare	324 (66.8)	26 (76.5)	71 (74.0)	92 (69.7)	7 (87.5)	
Sometimes	125 (25.8)	7 (20.6)	18 (18.8)	28 (21.2)	1 (12.5)	
Often	36 (7.4)	1 (2.9)	7 (7.3)	12 (9.1)	0 (0.0)	
Physical activity, N (%)						0.741
Rare	229 (47.2)	18 (52.9)	49 (51.0)	66 (50.0)	3 (37.5)	
Sometimes	195 (40.2)	13 (38.2)	36 (37.5)	44 (33.3)	3 (37.5)	
Regular	61 (12.6)	3 (8.8)	11 (11.5)	22 (16.7)	2 (25.0)	
Economic status, N (%)						0.541
Low	103 (21.2)	5 (14.7)	19 (19.8)	32 (24.2)	3 (37.5)	
Middle	174 (35.9)	9 (26.5)	29 (30.2)	47 (35.6)	2 (25.0)	
High	208 (42.9)	20 (58.8)	48 (50.0)	53 (40.2)	3 (37.5)	

BMI: body mass index; SBP: systolic blood pressure; ALT: alanine aminotransferase; DM: diabetes mellitus.

The findings of the Cox-PH models for the primary composite outcomes are presented in Table 2. Compared with the simvastatin group, the HRs (95% confidence intervals [CIs]) for the primary composite outcomes of the atorvastatin, pitavastatin, and rosuvastatin group were 0.875 (0.426–1.794), 1.238 (0.339–4.521), and 0.788 (0.267–2.323), respectively, after adjusting for age (Cox-PH model 1). After fully adjusting for age, smoking status, alcohol consumption, physical activity, BMI, SBP, total cholesterol, ALT, economic status, and DM, the HRs (95% CIs) of the atorvastatin, pitavastatin, and rosuvastatin groups were 0.956 (0.456–2.005), 1.347 (0.354–5.116), and 0.943 (0.317–2.803), respectively (Cox-PH model 3). In the pravastatin group, the number of outcome events was insufficient; thus, there were no statistically realistic results. The association between different statin types and the recurrence of CCVD events are shown in Table 3. Compared with the simvastatin group, after fully adjusting for confounders, the HRs (95% CIs) in the atorvastatin, pitavastatin, and rosuvastatin groups were 1.031 (0.479–2.220), 1.412 (0.366–5.449), and 1.031 (0.340–3.123), respectively.

**Table 2:** Cox-proportional hazard regression models for primary composite outcomes for each statin compared with simvastatin.

Model	Statin	HR (95% CI)
1	Atorvastatin	0.875 (0.426–1.794)
	Pitavastatin	1.238 (0.339–4.521)
	Rosuvastatin	0.788 (0.267–2.323)
	Pravastatin	N/A
2	Atorvastatin	0.900 (0.435–1.862)
	Pitavastatin	1.255 (0.342–4.603)
	Rosuvastatin	0.753 (0.254–2.232)
	Pravastatin	N/A
3	Atorvastatin	0.956 (0.456–2.005)
	Pitavastatin	1.347 (0.354–5.116)
	Rosuvastatin	0.943 (0.317–2.803)
	Pravastatin	N/A

HR: hazard ratio; CI: confidence interval.

Model 1: adjusted for age.

Model 2: adjusted for sex and smoking status, alcohol consumption and physical activity in addition to the age variable considered in Model 1.

Model 3: adjusted for body mass index, systolic blood pressure, total cholesterol, alanine aminotransferase, economic status, and diabetes, in addition to the variables considered in Model 2.

**Table 3:** Cox-proportional hazard regression models for the recurrence of cardio-cerebrovascular events for each statin compared with simvastatin.

Outcome	Statin	HR (95% CI)
Cardio-cerebrovascular events	Atorvastatin	1.031 (0.479–2.220)
	Pitavastatin	1.412 (0.366–5.449)
	Rosuvastatin	1.031 (0.340–3.123)
	Pravastatin	N/A

HR: hazard ratio; CI: confidence interval.

Adjusted for age, sex, smoking status, alcohol consumption, physical activity, body mass index, systolic blood pressure, total cholesterol, alanine aminotransferase, economic status, and diabetes.

**Fig 2:**
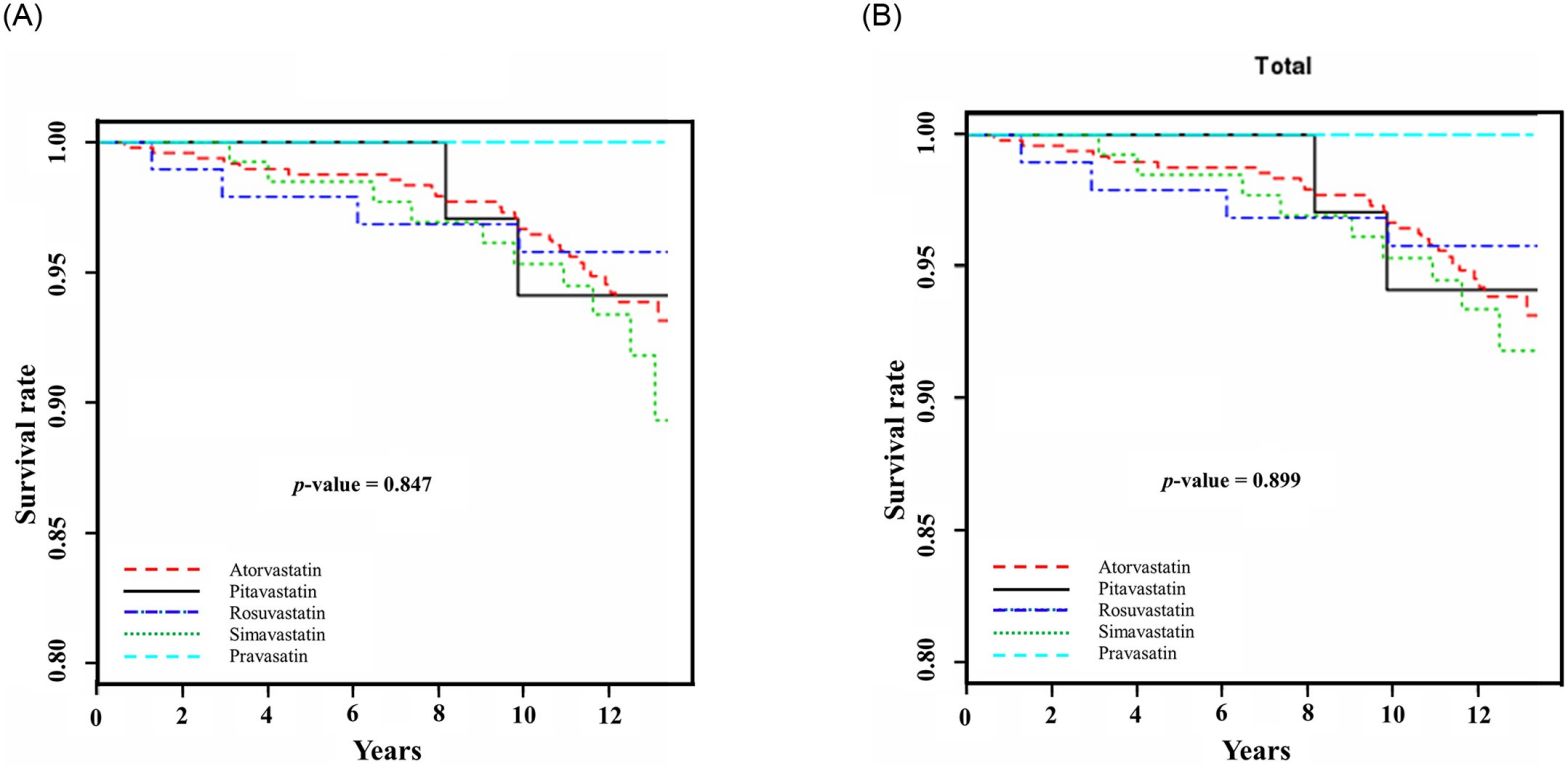
Kaplan-Meier estimates of primary composite outcomes and cardio-cerebrovascular events. A) Primary composite outcomes of all causes of death and/or recurrence of cardio-cerebrovascular disease (CCVD). B) Recurrence of cardio-cerebrovascular events.

The survival analysis was performed using the Kaplan-Meier method and log rank test to estimate the five statins’ effects on the primary composite outcomes and recurrence of CCVD events as indicated in Fig 2. There were no statistically significant differences between the different types of statin (p-values > 0.05).

## Discussion

The present study is a retrospective national cohort study that compared the efficacy of secondary prevention for CCVD among different types of statins using claim data from the NHIS in Korea. This study shows that there was no significant difference in preventing the recurrence of CCVD and/or death among five different types of statin in CCVD patients.

Statins were classified into three groups according to their reported LDL-cholesterol lowering intensity. The intensity will depend on the individual dose, but in general, atorvastatin, and rosuvastatin belong to moderate to high intensity groups, and the remaining statins are classified as low or moderate intensity groups [9]. The 2018 American College of Cardiology/American Heart Association guidelines recommend that patients with clinical atherosclerotic CCVD should reduce their LDL-cholesterol with either a high-intensity statin therapy or via a maximum tolerated statin therapy [10]. Due to their efficacy and safety, statins are widely administered for primary and secondary prevention treatment of ASCVD in individuals with dyslipidemia [3–6].

Different types of statins have different pharmacokinetics, as well as, varied clinical efficacies to improve patient lipid profiles and to prevent ASCVD [7]. In particular, the degree of LDL-cholesterol reduction achieved with statins appears different among Asian and Western patients. Asian CCVD patients often have an increased response to statins. As a result, recommended drug dosages often tend to be lower in Asian countries than in Western countries [11]. However, there is insufficient evidence to directly compare the efficacy of different statins in the secondary prevention of CCVD events [12]. In addition, it is known that individual statins have different efficacy on LDL-cholesterol reduction and HDL-cholesterol increase [13–15].

This study attempted to investigate whether there are differences in the secondary preventive efficacy of different types of statins. To maintain a homogeneous participant pool, we excluded subjects who were prescribed two or more statin types, or even one statin for ≤30 days. The results of this study show that there is no difference in the secondary prevention effect between the different types of statins. These results show that classification according to the intensity of LDL-cholesterol reduction for each statin may not have a significant difference in the effect of secondary CCVD prevention. This is probably due to a more complex mechanism besides the basic action of statins to reduce LDL-cholesterol [16, 17].

Statins inhibit HMG-CoA reductase activity in the mevalonate pathway. The mevalonate pathway produces mevalonic acids, which are precursors of cholesterol and some non-sterol isoprenoid derivatives. Isoprenoid derivatives play an important role in the regulation of various cellular functions including proliferation, differentiation, and survival [18, 19]. Statins inhibit the production of isoprenoid derivatives in the cholesterol pathway. Therefore, statins are known to be pluripotent in their ability to modulate cell signalling and to reduce oxidative stress and pro-inflammation [20]. In addition, since the data used in this study was obtained in a real-world setting, it is necessary to interpret the results in consideration of the respective conditions of the participants included in this study. For example, high-intensity statins may be prescribed to participants who are at a higher risk of CCVD, while low-intensity statins may be prescribed to participants with relatively low risk of CCVD. In addition, NHIS did not provide a data regarding the participants’ detailed lifestyle and behaviors, such as dietary patterns, which can affect the efficacy of secondary prevention, so these factors were not analyzed in this study. There may be a confounding effect on secondary prevention between different types of statin that was not accounted for in this study and future studies should consider dietary patterns in their analysis.

There are other limitations when interpreting the results of this study. First, several potentially confounding factors have been adjusted, however some residual confounding effects could not be completely controlled for in this study, and included lifestyle factors and/or underlying genetic or familial conditions. We also could not include the non-statin lipid-lowering agents as confounders due to the limited availability of data. Second, the main lipid target for the prevention of atherosclerotic CCVD is LDL cholesterol, but we were unable to include LDL cholesterol, triglycerides, and HDL cholesterol in our analysis. This is because the NHIS-HEALS cohort data has been provided with detailed lipid profile since 2009, so therefore the baseline data could not be acquired. Instead of LDL cholesterol, we adopted total cholesterol in our analysis. Third, since the operational definition of CCVD was determined based on the ICD-10^th^ codes, the participants in the study might not match actual CCVD patients in a real-world scenario. In addition, major adverse cardiovascular events (MACE) may not coincide precisely with the CCVD events that were operationally defined by the ICD-10^th^ codes in this study. Because the definition of MACE may vary from study to study [21], it may be difficult to accurately compare the result of this study with similar studies involving MACE. We defined the recurrence of CCVD with ICD-10^th^ codes I21-24, I63, I65, and I66 and/or all causes of death. Fourth, to maintain a homogeneous participant pool, we excluded subjects who were prescribed two or more statin types, or one statin for ≤30 days. Thus, the number of participants in the statin group in this study was small. Similarly, due to the small number of CCVD recurrences, it was not possible to analyze the effect of statins over time in detail. In addition, the sample size of the “Pravastatin” group is small, which may affect the statistical analysis. Therefore, we indicated “N/A” in the Tables 2 and 3. Large-scale clinical trials are required to compare the secondary preventive effects of each statin type on CCVD. Fifth, we could not guarantee that statin users took their medication as prescribed. Finally, selection bias and confounding by indication may exists because many participants were excluded based on inclusion and exclusion criteria.

Despite the several limitations, this study has several advantages. Of utmost importance, we used the national cohort data that represents Korea’s total population based on true measurements in clinical settings. In addition, since this study analyzed insurance claim data that included disease diagnosis, health and lifestyle questionnaires, blood tests, such as lipid profiling, and prescriptions, recall bias is minimized. Finally, regarding the effort to evaluate the differences in efficacy between different types of statins, all participants in this study took only one type of statin during a relatively long study period (median follow-up duration: 12.4 years). Thus, the long-term effect of secondary prevention was for each type of statin.

## Conclusion

In conclusion, in this Korean study, no significant differences were observed in the efficacies for preventing the recurrence of CCVD events and/or death according to different types of statins administered to CCVD patients. However, further large-scale clinical trials regarding the beneficial effects of secondary prevention of CCVD among individual statins are required.

## Supporting information

S1 Table Event count according to type of statin in CCVD patients. (DOCX)
