# Supplementary material for: Comparing different types of statins for secondary prevention of cardio-cerebrovascular disease from a national cohort study
Source: PLoS One. 2021 Feb 25;16(2):e0247419. doi: 10.1371/journal.pone.0247419 (PMC7906327; doi:10.1371/journal.pone.0247419)
Supplement: S1 Table — (DOCX) [file pone.0247419.s001.docx]

**S1 Table**. Event count according to type of statin in CCVD patients

| Number (%) | Simvastatin  (N=132) | Atorvastatin  (N=485) | Pitavastatin  (N=34) | Rosuvastatin  (N=96) | Pravastatin  (N=8) |
| --- | --- | --- | --- | --- | --- |
| Cardio-cerebrovascular events | 10 (7.58) | 30 (6.19) | 3 (8.82) | 5 (5.21) | 0 |
| Cardiovascular events | 2 (1.52) | 6 (1.24) | 0 | 2 (2.08) | 0 |
| Cerebrovascular events | 7 (5.30) | 24 (4.95) | 3 (8.82) | 3 (3.13) | 0 |
| Death | 1 (0.76) | 0 | 0 | 0 | 0 |
